# Supplementary material for: Human Neocortical Neurosolver (HNN), a new software tool for interpreting the cellular and network origin of human MEG/EEG data
Source: eLife. 2020 Jan 22;9:e51214. doi: 10.7554/eLife.51214 (PMC7018509; doi:10.7554/eLife.51214)
Supplement: Transparent reporting form [file elife-51214-transrepform.docx]

***eLife’s* transparent reporting form**

We encourage authors to provide detailed information *within their submission* to facilitate the interpretation and replication of experiments. Authors can upload supporting documentation to indicate the use of appropriate reporting guidelines for health-related research (see EQUATOR Network), life science research (see the [BioSharing Information Resource](https://biosharing.org/)), or the [ARRIVE guidelines](http://www.plosbiology.org/article/info:doi/10.1371/journal.pbio.1000412) for reporting work involving animal research. Where applicable, authors should refer to any relevant reporting standards documents in this form.

If you have any questions, please consult our Journal Policies and/or contact us: [editorial@elifesciences.org](mailto:editorial@elifesciences.org).

**Sample-size estimation**

- You should state whether an appropriate sample size was computed when the study was being designed
- You should state the statistical method of sample size computation and any required assumptions
- If no explicit power analysis was used, you should describe how you decided what sample (replicate) size (number) to use

Please outline where this information can be found within the submission (e.g., sections or figure legends), or explain why this information doesn’t apply to your submission:

This information does not apply to our Tools & Resources submission because we are primarily describing the design and use of our new Human Neocortical Neurosolver (HNN) software platform which allows clinicians/experimentalists to perform detailed biophysical simulations of a validated thalamocortical circuit model that generates the most commonly observed patterns (event-related potentials, brain rhythms) in source-localized human MEG activity. HNN’s model has been validated in an extensive set of peer-reviewed publications using invasive electrophysiological recordings from multiple species (mice, rats, nonhuman primates) and experimental conditions (attention tasks during tactile stimulation, spontaneous ongoing activity, etc.).

- You should include a definition of biological versus technical replication
- The data obtained should be provided and sufficient information should be provided to indicate the number of independent biological and/or technical replicates
- If you encountered any outliers, you should describe how these were handled
- Criteria for exclusion/inclusion of data should be clearly stated
- High-throughput sequence data should be uploaded before submission, with a private link for reviewers provided (these are available from both GEO and ArrayExpress)

Please outline where this information can be found within the submission (e.g., sections or figure legends), or explain why this information doesn’t apply to your submission:

This information does not apply to our submission for the reasons stated above.

- Statistical analysis methods should be described and justified
- Raw data should be presented in figures whenever informative to do so (typically when N per group is less than 10)
- For each experiment, you should identify the statistical tests used, exact values of N, definitions of center, methods of multiple test correction, and dispersion and precision measures (e.g., mean, median, SD, SEM, confidence intervals; and, for the major substantive results, a measure of effect size (e.g., Pearson's r, Cohen's d)
- Report exact p-values wherever possible alongside the summary statistics and 95% confidence intervals. These should be reported for all key questions and not only when the p-value is less than 0.05.

Please outline where this information can be found within the submission (e.g., sections or figure legends), or explain why this information doesn’t apply to your submission:

Statistical analysis methods are described in: **1. Methods**, in the Exogenous Driving Inputs section, which describes the synaptic inputs (timing, strength, temporal distribution), and how the HNN platform allows users to specify these inputs. Related information/examples are presented in the legends of figure 4, 7, 8, 9, and 11; **2.** In **Results** in Tutorials on ERPs and low-frequency oscillations, and Example model optimization for the suprathreshold sensory evoked response data set. **3.** In **Methods**, in the ERP optimization tools description. **4. In Supplementary Materials**, in Sensitivity Analyses of ERP Simulations.

**Group allocation**

- Indicate how samples were allocated into experimental groups (in the case of clinical studies, please specify allocation to treatment method); if randomization was used, please also state if restricted randomization was applied
- Indicate if masking was used during group allocation, data collection and/or data analysis

Please outline where this information can be found within the submission (e.g., sections or figure legends), or explain why this information doesn’t apply to your submission:

Group allocation is not applicable to this submission. Although we describe different uses of HNN’s model for simulating event-related potentials and brain rhythms in different conditions (for example, in the Results section ***Tutorials on ERPs and low-frequency oscillations****),* these simulations have been described and validated in our prior publications, as referenced.

- Where provided, these should be in the most useful format, and they can be uploaded as “Source data” files linked to a main figure or table
- Include model definition files including the full list of parameters used
- Include code used for data analysis (e.g., R, MatLab)
- Avoid stating that data files are “available upon request”

Please indicate the figures or tables for which source data files have been provided:

Our Human Neocortical Neurosolver tutorials and full documentation are provided on our website ([https://hnn.brown.edu](https://hnn.brown.edu/)). All source code, example experimental data, and model parameter files referenced in the submission are available on the public github page (<https://github.com/jonescompneurolab/hnn>). Parameters for the sensitivity analyses that produced values in Supplementary Table 1 are described in Supplementary Materials, Sensitivity Analyses of ERP Simulations.

We have included a shortened description of the template model in the Methods section, with tables (Table 1 and Table 2 in the methods) for the critical model parameters.

The supplementary figures and tables are also provided for more comprehensive explanation of the ERP optimization algorithm.
